# Hunger and housing: Economic disparities in current and daily tobacco use among high school students in the United States in 2021

**DOI:** 10.1016/j.pmedr.2024.102901

**Published:** 2024-10-18

**Authors:** Dale S. Mantey, Kathryn M. Janda-Thomte, Adam C. Alexander, Onyinye Omega-Njemnobi, Steven H. Kelder

**Affiliations:** a UTHealth, University of Texas School of Public Health, Department of Health Promotion and Behavioral Science, Austin, TX, United States; b UTHealth, University of Texas School of Public Health, Department of Epidemiology, Human Genetics & Environmental Sciences, Austin, TX, United States; c Michael and Susan Dell Center for Healthy Living, Austin, TX, United States; d Department of Public Health, Robbins College, Baylor University, Waco, TX, United States; e TSET Health Promotion Research Center, Stephenson Cancer Center, the University of Oklahoma Health Science Centers, Oklahoma City, OK, United States; f Department of Family and Preventive Medicine, College of Medicine, University of Oklahoma Health Sciences Center, Oklahoma City, OK, United States

**Keywords:** Economic inequalities, Youth, Tobacco use, Daily use, Addiction, Homelessness, Food insecurity, Social determinants of health

## Abstract

**Introduction:**

Economic disparities in tobacco use and dependence are well-documented among adults but not adolescents. This study aims to examine economic disparities in patterns of tobacco use among a nationally representative sample of high school students in Spring 2021.

**Methods:**

We analyzed data from n = 6750 US high school student via the 2021 Adolescent Behaviors and Experiences Survey (ABES). We estimated the association between experiencing homelessness and food insecurity (analyzed independently) and current (past 30-day) and daily (all 30 days) use of four tobacco products (e-cigarettes; cigarettes; cigars; smokeless). Models controlled for sex, race/ethnicity, age, and sexual identity.

**Results:**

Overall, ∼1.8 % experienced homelessness and 23.7 % experienced food insecurity. Experiencing homeless was significantly associated with greater odds of current use for e-cigarette (aOR: 3.43), cigarettes (aOR: 5.58), cigars (aOR: 10.47), and smokeless tobacco (aOR: 4.41) as well as greater risk for daily use of e-cigarettes (aOR: 2.66), cigarettes (aOR: 10.94), and cigars (aOR: 5.23) but not smokeless tobacco (aOR: 2.48; 95 %CI: 0.51–12.16). Food insecurity was significantly associated with greater odds of current use of e-cigarettes (aOR: 2.00), cigarettes (aOR: 2.15), and cigars (aOR: 2.44) but not smokeless (aOR: 1.04; 95 % CI: 0.56–1.93). No association was observed between food insecurity and daily tobacco use.

**Conclusion:**

Substantial economic disparities in tobacco use were observed in a nationally representative sample of high school students. Interventions should consider prioritizing economic determinants of health during adolescence, including a focus on preventing youth tobacco use as well as addressing upstream determinants of homelessness and food insecurity.

## Highlights

• Approximately 1.8 % of high school students experienced homelessness in 2021.
• Approximately 23.7 % of high school students experienced food insecurity in 2021.
• Youth experiencing homelessness were more likely to report current and daily use of cigarettes, cigars, and e-cigarettes.
• Findings are consistent with economic disparities in adult tobacco use.
• Economic disparities should be further emphasized in health disparities research.

## 1 Introduction

In the United States, approximately 480,000 individuals die each year due to tobacco use (U.S. Department of Health and Human Services, 2014). Most adults start using tobacco during adolescence (U.S. Department of Health and Human Services, 2014) and tobacco use during adolescence presents several public health concerns, including increased susceptibility to nicotine, a highly addictive stimulant, and neurotoxin to developing brains (Benowitz, 2010, Benowitz et al., 2017). Behavioral indicators of nicotine dependence, including frequent or daily use, (Mantey et al., 2021, Mantey et al., 2019) are common among youth in the US. Prior to the COVID-19 pandemic (2019), national estimates show a considerable proportion of those who use e-cigarettes (30.4 %), combustible cigarettes (28.9 %), and smokeless tobacco (34.1 %) report use of those products on 20+ days per month (Wang et al., 2019). Understanding the patterns and correlates of nicotine dependence among adolescents who use tobacco is critical to developing evidence-based interventions and regulatory policy.

Economic factors (e.g., income and health insurance coverage) are among strongest and most consistent correlates of tobacco use among adults (Cornelius et al., 2023, Cornelius et al., 2022). The tobacco industry spends billions per year advertising their products in low-income communities (Glasser and Roberts, 2021), with price discounts at the point-of-sale (POS) being the most well-funded promotional tactic (Ma et al., 2022). As a result, youth from low-income backgrounds are exposed to a greater concentration of tobacco advertising and promotions in their environment via POS marketing (Glasser and Roberts, 2021) as well as a greater social normalization of tobacco use via higher prevalence adult tobacco use (Cornelius et al., 2023, Cornelius et al., 2022). Adolescent tobacco use is heavily influenced by social and ecological influences, particularly adult tobacco use and marketing exposure (Prevention NCfCD, 2012); however, despite the concentration of risk factors for tobacco use in low-income environments, minimal research has investigated the association between the economic condition and adolescent tobacco use in national samples.

Homelessness (Dworsky, 2020) and food insecurity (Jones et al., 2023) are economic inequalities experienced by millions of youth in the US. Research has characterized the high prevalence of tobacco use and nicotine dependence among youth experiencing homelessness (Patterson et al., 2022, Tucker et al., 2020), reflecting a similar correlation between economic conditions and tobacco use observed in national surveillance of adults (Cornelius et al., 2023, Cornelius et al., 2022). However, prior investigations of tobacco use among youth experiencing homelessness and/or food insecurity have been restricted to small cohorts (Patterson et al., 2022, Tucker et al., 2020, Kim-Mozeleski and Pandey, 2020), necessitating further study among generalizable samples (Robson et al., 2017). We will expand on these prior studies by investigating the association between indicators of economic condition and adolescent tobacco use using a nationally representative sample.

The COVID-19 pandemic disrupted the economic security of millions in the United States (Yeboah et al., 2021, Gundersen et al., 2021). Household food insecurity remained steady at 10.5 % at the national level from 2019 to 2020, yet food insecurity significantly increased among households with children from 13.6 % in 2019 to 14.8 % in 2020 according to United States Department of Agriculture data (Coleman-Jensen et al., 2022). There is limited data available on the prevalence of food insecurity specifically among adolescents before or during the COVID-19 pandemic, given that food insecurity prevalence is often only reported among all children under 18 or specifically young children (aged 5 and under) (Coleman-Jensen et al., 2022, Damages SPSTY, 0000). However, a few studies are using nationally representative data collected in 2021 that found food insecurity prevalence among adolescents was higher than the national prevalence (Krause et al., 2022); however, no national prevalence data for this age group is widely available. To our knowledge, national estimates of changes in homelessness among youth during the COVID-19 pandemic are also unavailable (Dworsky, 2020).

### 1.1 Study aims & hypotheses

We examine economic disparities in tobacco use among a nationally representative sample of high school students in spring 2021. Economic disparities were examined via two self-reported measures: experiencing homelessness in the past 30 days and experiencing food insecurity during the COVID-19 pandemic. We examined current and frequent use of four (4) tobacco products: (1) e-cigarettes; (2) cigarettes; (3) cigars; and (4) smokeless tobacco. First, we explored any use of each product in the past 30 days (i.e., current use) among all high school students. Second, we compared economic disparities in risk for daily use (i.e., 30 days/month) among those who currently use each product. We hypothesize that both measures of economic disparities will be statistically associated with greater odds of current and daily use of each product. Findings from this study will contribute to our understanding of economic disparities in adolescent tobacco use in the US.

## 2 Methods

### 2.1 Data set & study sample

We analyzed data from the Adolescent Behaviors and Experiences Survey (ABES), a cross-sectional survey collected by the Center for Disease Control and Prevention (CDC) during the Spring 2021 semester (January to June). ABES used a three-stage, cluster probability-based sampling approach to produce nationally representative estimates for 9th to 12th-grade students (i.e., high school) in public and private schools in the US in 2021. Data were collected via an online survey, and the survey was designed to assess student behaviors and experiences during the COVID-19 pandemic. The methodology, protocol, and survey instrument for ABES mirrors that of the Youth Risk Behavioral Surveillance Survey (YRBSS). The CDC published the Study design, sampling, and procedures for ABES and YRBS elsewhere (Rico, 2021). All data were de-identified and made publicly available by the CDC and, as such, the study is exempt from internal review board.

The ABES dataset has data for n = 7705 high school students. Per CDC recommendations, we removed n = 169 participants who responded, “I do not know what this question is asking,” when asked to self-report their sexual identity. An additional n = 786 participants (10.3 % of the weighted sample) had incomplete data on the independent variables used in this analysis. Next, for each analysis, we excluded participants with missing data on the dependent variable (i.e., tobacco use) from the n = 6750 eligible participants. This resulted in the following analytic samples, corresponding to each outcome of interest: (1) n = 6342 for e-cigarette use; (2) n = 6719 for combustible cigarettes; (3) n = 6651 for combustible cigars; and (4) n = 6717 for smokeless tobacco. Methodological and analytic discussion of missingness and non-response are described below.

### 2.2 Measures

*Economic Disparities.* This study used two primary independent variables to investigate economic disparities. First, we examined self-reported measures of experiencing homelessness. Participants were asked: “During the past 30 days, where did you usually sleep?” Responses included: (1) In my parent’s or guardian’s home; (2) “In the home of a friend, family member, or other person because I had to leave my home or my parents or guardian cannot afford housing”; (3) “In a shelter or emergency housing”; (4) “In a motel or hotel”; (5) “In a car, park, campground, or other public place”; (6) “I do not have a usual place to sleep”; and (7) “Somewhere else.” Consistent with CDC guidelines, youth were considered to be experiencing homelessness if they responded with 2 through 6; youth who responded with 1 or 7 were considered not experiencing homelessness.

Additionally, we examined self-reported measures of food insecurity. Participants were asked: “During the COVID-19 pandemic, how often did you go hungry because there was not enough food in your home?” Consistent with CDC guidelines, participants who responded “never” were considered to not be experiencing food insecurity.

*Current Use.* The first outcome variable examined for this study was the past 30-day use of four tobacco products: e-cigarettes, combustible cigarettes, combustible cigars, and smokeless tobacco. Use of each tobacco product in the past 30 days (*current use*) was assessed and examined independently. To assess tobacco use, participants were asked the following question for each tobacco product category: “During the past 30 days, how many days did you use [tobacco product]?” Possible responses ranged from 0 days (non-user) through “All 30 Days”. Participants who reported using the product, even once, during the past 30 days were considered to currently use that product.

*Daily Use*. The second outcome variable for this study was daily use among those who reported past 30-day use of each product. This outcome was categorized as infrequent and frequent tobacco use based on the number of days that they reported using each product within the past 30 days. We categorized individuals who reported using on 1–29 days as infrequent and “all 30 days” as daily use. The comparison of interest is non-daily relative to the daily use of each product, respectively.

*Covariates*. This study controlled for several demographic variables. Sex assigned at birth was coded as a binary variable, and males served as the referent. Race/ethnicity was categorized as non-Hispanic White (referent), Hispanic/Latino, Non-Hispanic Black, and non-Hispanic “Other” which reflects those who identified as American Indian/Alaskan Native, Asian, Native Hawaiian/Other Pacific Islander, or Multi-Racial, but did not identify as Hispanic/Latino. These categories were selected in order to be consistent with the Federal Interagency Technical Working Group on Race and Ethnicity Standard for revising the Office of Management and Budget (OMB) 1997 Statistical Policy Directive No. 15: Standards for Maintaining, Collecting, and Presenting Federal Data on Race and Ethnicity (Register, 2023). Age was coded categorically, reflecting 18+ (referent; coded as 0), 17 years old, 16 years old, 15 years old, and 14 or younger (coded as 4). We also controlled for sexual identity, which was categorized as (1) heterosexual (referent); (2) gay or lesbian; (3) bisexual; and (4) and “I describe my sexual identity some other way” or questioning.

### 2.3 Missingness and Non-Response

We conducted a complete case analysis (CCA) with the final analytic samples varying by missingness on each dependent variable; we elected this procedure for several reasons. First, the ABES survey procedures allowed partnering schools (i.e., where student participants) to remove as much as one-third of the survey questions (Brener et al., 2013), introducing the possibility of systematic patterns of missingness (i.e., missing not at random [MNAR]) entirely removed from the participant. We investigated patterns of nonresponse and found that of the n = 786 participants with missing data for the independent variables. In general, multiple imputation is inappropriate for data MNAR (Sterne et al., 2009). Furthermore, combined with the size of our dataset (Henry et al., 2013) and the categorical coding of our variables (Allison, 2005), our examination of missingness suggests using a complete case analysis to minimize bias due to nonresponse.

### 2.4 Statistical analyses

Descriptive statistics with 95 % confidence intervals were estimated to capture participant characteristics. Our investigation of experiencing homelessness and food insecurity was conducted independently. First, we conducted four multivariable logistic regression analyses to estimate the association between experiencing homelessness and past 30-day use of: (1) e-cigarettes, (2) combustible cigarettes, (3) combustible cigars, and (4) smokeless tobacco. Second, we conducted four multivariable, multinomial logistic regression analyses to estimate the relationship between daily (30 of 30 days) tobacco use, relative to non-daily tobacco use, among youth experiencing homelessness for each product. For these multinomial logistic regressions, we compared daily to non-daily use; however, non-use was retained as a category in the analytic model to maintain the national weighting and sampling frame of the dataset. Second, we replicated these models using food insecurity as the primary independent variable; all other parameters of the statistical model were retained.

All analyses controlled for sex assigned at birth, race/ethnicity, grade, and sexual identity. Data were weighted to be nationally representative and to account for the complex sampling design, using the svy and svy,subpop() commands. Analyses were conducted in Stata 17.0 (College Station, TX).

## 3 Results

### 3.1 Descriptive statistics

Approximately 1.8 % of youth reported experiencing homelessness, and 23.7 % reported food insecurity. As seen in Table 1, the prevalence of current use was 15.4 % for e-cigarettes, 3.2 % for cigarettes, 2.0 % for cigars, and 1.6 % for smokeless tobacco. As seen in Table 2, prevalence of daily use was 31.5 % for e-cigarettes, 9.8 % for cigarettes, 13.1 % for cigars, and 17.5 % of smokeless tobacco.

**Table 1:** Descriptive statistics of current (past 30-day) use of E-cigarettes, cigarettes, cigars, and smokeless tobacco among high school Students in the United States, Spring 2021; Adolescent Behaviors and Experiences Survey (ABES).

	Current^a^ Electronic Cigarette Users N = 6,342	Current^a^ Cigarette Smokers N = 6,651	Current^a^ Cigar Smokers N = 6,717	Current^a^ Smokeless Tobacco Users N = 6,719
	Percent (95 % CI)			
Total Prevalence	15.4 (13.0–18.1)	3.2 (2.4–4.4)	2.0 (1.6–2.6)	1.6 (1.1–2.4)

Homelessness^b^				
No	15.0 (12.7–17.7)	3.1 (2.2–4.2)	1.8 (1.4–2.3)	1.4 (1.0–2.2)
Yes	37.3 (24.8–51.8)	15.9 (8.7–27.3)	17.4 (10.3–28.0)	12.1 (6.3–21.8)

Food Insecurity^c^				
No	13.5 (11.3–16.1)	2.7 (2.0–3.7)	1.5 (1.1–2.1)	1.7 (1.1–2.6)
Yes	21.5 (17.0–26.8)	5.0 (3.2–7.6)	3.6 (2.5–5.1)	1.4 (0.9–2.2)

Sex				
Male	14.1 (11.9–16.6)	3.4 (2.5–4.8)	2.8 (2.0–3.8)	2.8 (1.8–4.1)
Female	16.6 (13.2–20.5)	3.0 (2.1–4.5)	1.3 (0.9–1.9)	0.5 (0.3–1.0)

Race/Ethnicity^d^				
Non-Hispanic, White	20.2 (17.1–23.7)	4.8 (3.6–6.4)	2.2 (1.5–3.2)	2.6 (1.8–3.8)
Hispanic/Latino	9.8 (7.2–13.3)	1.8 (1.1–3.0)	1.1 (0.7–1.7)	0.6 (0.3–1.2)
Non-Hispanic, Black	10.2 (7.8–13.2)	0.6 (0.1–2.6)	2.5 (1.5–4.1)	0.3 (0.01–0.09)
Non-Hispanic, Other	11.2 (8.0–15.6)	2.2 (1.1–4.5)	2.9 (1.5–5.5)	0.7 (0.3–1.6)

Age				
18 or older	19.2 (15.7–23.4)	4.9 (2.9–8.1)	2.9 (1.8–4.6)	2.3 (0.9–5.6)
17 years old	19.9 (16.3–24.0)	4.3 (2.7–6.8)	2.8 (1.8–4.3)	2.4 (1.5–3.7)
16 years old	14.5 (12.0–17.5)	2.6 (1.6–4.2)	1.6 (1.0–2.7)	1.6 (0.9–2.9)
15 years old	12.7 (9.4–17.0)	2.9 (1.7–5.1)	1.8 (1.0–3.2)	1.1 (0.6–2.0)
14 years old	9.1 (6.7–12.1)	1.0 (0.5–2.0)	0.7 (0.4–1.3)	0.4 (0.2–0.9)

Sexual Identity^e^				
Heterosexual	14.5 (12.0–17.3)	2.9 (2.1–4.0)	1.9 (1.4–2.6)	1.9 (1.2–2.8)
Gay/Lesbian	16.6 (10.9–24.6)	4.8 (2.0–10.8)	3.4 (1.6–7.2)	1.7 (0.6–4.3)
Bisexual	21.7 (17.2–27.1)	5.8 (3.3–9.9)	3.2 (1.8–5.7)	0.9 (0.3–2.3)
Questioning/Other	15.5 (11.5–20.6)	3.0 (1.6–5.6)	0.9 (0.5–1.9)	0.4 (0.2–0.9)

**NOTE:** Figures are reported to be interpreted as: “15% of youth not experiencing homelessness used e-cigarettes while 37.3% of youth who were experiencing homelessness used e-cigarettes, in the past 30-days”.

a Current reflects past 30-day use of each product.

b Participants were asked: “During the past 30 days, where did you usually sleep?”

c Yes reflects not reporting “never” to the question: “ During the COVID-19 pandemic, how often did you go hungry because there was not enough food in your home?”

d For race/ethnicity, Hispanic ethnicity does not consider participant response to question of racial category. Non-Hispanic “Other” reflects those who identified as American Indian/Alaskan Native, Asian, Native Hawaiian/Other Pacific Islander, or Multi-Racial, but did not identify as Hispanic/Latino. For this “NH” is “non-Hispanic.”

e Questioning includes responses: “I describe my sexual identity some other way” or “I am not sure about my sexual identity (questioning)”.

**Table 2:** Descriptive statistics of daily use of E-cigarettes, cigarettes, cigars, and smokeless tobacco among current users of each product among high school students in the United States, Spring 2021; Adolescent Behaviors and Experiences Survey (ABES).

	Daily^a^ Electronic Cigarette Users N = 1,023	Daily^a^ Cigarette Smokers N = 225	Daily^a^ Cigar Smokers N = 163	Daily^a^ Smokeless Tobacco Users N = 124
	Percent (95 % CI)			
Total Prevalence	31.5 (26.1–37.5)	9.8 (6.6–14.4)	13.1 (7.6–21.7)	17.5 (10.8–27.0)

Homelessness^b^				
No	30.7 (25.3–30.7)	7.0 (4.0–12.1)	9.6 (5.2–17.2)	15.4 (8.0–27.8)
Yes	52.9 (35.0–70.1)	47.4 (25.2–70.7)	35.3 (15.7–61.6)	32.5 (10.8–65.8)

Food Insecurity^c^				
No	29.3 (23.4–36.1)	8.1 (4.7–13.7)	10.3 (4.2–23.0)	13.9 (6.7–26.5)
Yes	36.2 (28.6–44.6)	12.9 (7.7–20.9)	17.1 (8.4–31.7)	31.7 (14.1–56.8)

Sex				
Male	31.2 (24.6–38.6)	13.4 (8.0–21.6)	11.7 (5.0–25.0)	18.4 (11.1–28.9)
Female	31.8 (26.1–38.1)	6.1 (2.5–14.3)	16.0 (7.8–30.2)	13.0 (5.1–29.7)

Race/Ethnicity^d^				
Non-Hispanic, White	37.3 (31.3–43.8)	8.1 (4.8–13.6)	11.1 (4.6–24.3)	16.9 (9.3–28.7)
Hispanic/Latino	18.7 (12.5–27.0)	14.2 (6.5–28.4)	14.2 (5.7–31.4)	19.2 (6.0–46.8)
Non-Hispanic, Black	13.1 (6.4–24.7)	14.2 (12.2–67.0)	24.4 (10.3–473.6)	29.7 (7.8–68.1)
Non-Hispanic, Other	27.0 (14.4–44.9)	16.8 (5.5–41.1)	8.8 (2.3–28.3)	17.7 (4.1–51.6)

Age				
18 or older	40.7 (30.4–51.9)	12.1 (4.4–29.4)	12.7 (4.5–31.2)	28.8 (12.6–53.3)
17 years old	41.8 (33.3–50.9)	6.0 (2.3–14.6)	15.4 (7.3–29.5)	20.6 (8.6–41.9)
16 years old	25.4 (20.0–31.9)	11.1 (4.0–27.2)	12.4 (3.6–34.8)	10.8 (2.9–33.5)
15 years old	19.8 (11.6–31.7)	11.7 (3.7–31.1)	9.9 (2.4–33.1)	6.8 (1.5–26.5)
14 years old	18.0 (8.3–34.7)	12.3 (1.6–54.4)	17.5 (2.4–64.3)	14.6 (2.6–52.0)

Sexual Identity^e^				
Heterosexual	30.6 (25.5–36.3)	9.5 (5.7–15.4)	11.1 (5.7–20.5)	16.2 (9.1–27.0)
Gay/Lesbian	18.6 (8.1–37.0)	23.4 (7.3–59.5)	16.8 (4.0–49.4)	42.9 (10.3–83.1)
Bisexual	33.9 (21.0–49.8)	7.4 (2.4–20.8)	14.9 (4.7–38.4)	7.6 (1.1–38.5)
Questioning/Other	39.6 (23.2–58.7)	9.7 (2.2–33.3)	37.5 (13.7–69.4)	59.1 (24.6–86.5)

**NOTE:** Figures are reported to be interpreted as: “Among current e-cigarette users, prevalence of daily use was 30.7% among youth not experiencing homelessness and 52.9% among youth who were experiencing homelessness.”

a Daily reflects self-reported use of the corresponding product on 30 of the past 30 days.

b Participants were asked: “During the past 30 days, where did you usually sleep?”

c Yes reflects not reporting “never” to the question: “ During the COVID-19 pandemic, how often did you go hungry because there was not enough food in your home?”

d For race/ethnicity, Hispanic ethnicity does not consider participant response to question of racial category. Non-Hispanic “Other” reflects those who identified as American Indian/Alaskan Native, Asian, Native Hawaiian/Other Pacific Islander, or Multi-Racial, but did not identify as Hispanic/Latino. For this “NH” is “non-Hispanic.”

e Questioning includes responses: “I describe my sexual identity some other way” or “I am not sure about my sexual identity (questioning)”.

### 3.2 Current tobacco use

As seen in Table 3, experiencing homeless was significantly associated with greater odds of current use for e-cigarette (aOR: 3.43; 95 % CI: 1.81–6.48), cigarettes (aOR: 5.58; 95 % CI: 2.33–13.34), cigars (aOR: 10.47; 95 % CI: 5.41–20.28), and smokeless tobacco (aOR: 4.41; 95 % CI: 3.03–23.39), controlling for covariates.

**Table 3:** Association between experiencing homelessness and current (past 30-day) tobacco use among high school students in the United States, Spring 2021; Adolescent Behaviors and Experiences Survey (ABES).

	Current^a^ Electronic Cigarette Users N = 6,342	Current^a^ Cigarette Smokers N = 6,651	Current^a^ Cigar Smokers N = 6,717	Current^a^ Smokeless Tobacco Users N = 6,719
	Adjusted Odds Ratio (95 % CI)			
Homelessness^b^				
No	1.00 (Referent)	1.00 (Referent)	1.00 (Referent)	1.00 (Referent)
Yes	3.43 (1.81–6.48)	5.58 (2.33–13.34)	10.47 (5.41–20.28)	8.41 (3.03–23.39)

Sex				
Male	1.00 (Referent)	1.00 (Referent)	1.00 (Referent)	1.00 (Referent)
Female	1.19 (0.89–1.59)	0.82 (0.54–1.24)	0.47 (0.28–0.77)	0.23 (0.12–0.43)

Race/Ethnicity^c^				
Non-Hispanic, White	1.00 (Referent)	1.00 (Referent)	1.00 (Referent)	1.00 (Referent)
Hispanic/Latino	0.44 (0.31–0.61)	0.38 (0.23–0.63)	0.52 (0.30–0.91)	0.25 (0.13–0.48)
Non-Hispanic, Black	0.45 (0.31–0.65)	0.12 (0.03–0.55)	1.16 (0.57–2.37)	0.10 (0.03–0.31)
Non-Hispanic, Other	0.48 (0.32–0.70)	0.44 (0.22–0.88)	1.41 (0.68–2.93)	0.27 (0.12–0.60)

Age				
18 or older	1.00 (Referent)	1.00 (Referent)	1.00 (Referent)	1.00 (Referent)
17 years old	1.09 (0.79–1.51)	0.95 (0.50–1.82)	1.06 (0.59–1.91)	1.34 (0.49–3.63)
16 years old	0.74 (0.55–0.99)	0.55 (0.25–1.21)	0.57 (0.28–1.17)	0.79 (0.25–2.46)
15 years old	0.64 (0.45–0.89)	0.63 (0.89–1.38)	0.68 (0.29–1.56)	0.53 (0.16–1.81)
14 years old	0.45 (0.32–0.64)	0.23 (0.09–0.58)	0.29 (0.14–0.59)	0.22 (0.09–0.55)

Sexual Identity^d^				
Heterosexual	1.00 (Referent)	1.00 (Referent)	1.00 (Referent)	1.00 (Referent)
Gay/Lesbian	1.10 (0.61–1.97)	1.60 (0.62–4.14)	1.74 (0.84–3.58)	1.07 (0.37–3.07)
Bisexual	1.60 (1.14–2.23)	2.29 (1.32–4.31)	2.21 (1.02–4.83)	0.75 (0.24–2.33)
Questioning/Other	1.04 (0.81–1.33)	1.10 (0.56–2.16)	0.58 (0.24–1.38)	0.27 (0.09–0.77)

**NOTE:** Sex, race/ethnicity, age, and sexual identity were used as covariates to estimate adjusted odds ratios.

a Current reflects past 30-day use of each product.

b Binary indicator of self-reporting homelessness. Participants were asked: “During the past 30 days, where did you usually sleep?”

c For race/ethnicity, Hispanic ethnicity does not consider participant response to question of racial category. Non-Hispanic “Other” reflects those who identified as American Indian/Alaskan Native, Asian, Native Hawaiian/Other Pacific Islander, or Multi-Racial, but did not identify as Hispanic/Latino. For this “NH” is “non-Hispanic.”

d Questioning includes responses: “I describe my sexual identity some other way” or “I am not sure about my sexual identity (questioning)”.

Similarly, food insecurity was associated with significantly greater odds of current use of e-cigarettes (aOR: 2.00; 95 % CI: 1.51–2.66), cigarettes (aOR: 2.15; 95 % CI: 1.37–3.36), cigars (aOR: 2.44; 95 % CI: 1.58–3.77) but not smokeless tobacco (aOR: 1.04; 95 % CI: 0.56–1.93), controlling for covariates. These results are presented in Table 4.

**Table 4:** Association between food insecurity and current (past 30-day) tobacco use among high school students in the United States, Spring 2021; Adolescent Behaviors and Experiences Survey (ABES).

	Current^a^ Electronic Cigarette Users N = 6,342	Current^a^ Cigarette Smokers N = 6,651	Current^a^ Cigar Smokers N = 6,717	Current^a^ Smokeless Tobacco Users N = 6,719
	Adjusted Odds Ratio (95 % CI)			
Food Insecurity^b^				
No	1.00 (Referent)	1.00 (Referent)	1.00 (Referent)	1.00 (Referent)
Yes	2.00 (1.51–2.66)	2.15 (1.37–3.36)	2.44 (1.58–3.77)	1.04 (0.56–1.93)

Sex				
Male	1.00 (Referent)	1.00 (Referent)	1.00 (Referent)	1.00 (Referent)
Female	1.18 (0.89–1.56)	0.78 (0.51–1.20)	0.42 (0.26–0.68)	0.21 (0.11–0.38)

Race/Ethnicity^c^				
Non-Hispanic, White	1.00 (Referent)	1.00 (Referent)	1.00 (Referent)	1.00 (Referent)
Hispanic/Latino	0.40 (0.29–0.57)	0.35 (0.21–0.60)	0.47 (0.27–0.84)	0.25 (0.13–0.48)
Non-Hispanic, Black	0.40 (0.27–0.59)	0.11 (0.02–0.51)	1.04 (0.51–2.13)	0.10 (0.03–0.34)
Non-Hispanic, Other	0.43 (0.29–0.64)	0.38 (0.19–0.76)	1.14 (0.53–2.43)	0.25 (0.12–0.56)

Age				
18 or older	1.00 (Referent)	1.00 (Referent)	1.00 (Referent)	1.00 (Referent)
17 years old	1.07 (0.78–1.49)	0.92 (0.48–1.75)	1.01 (0.57–1.77)	1.29 (0.52–3.21)
16 years old	0.73 (0.54–0.99)	0.53 (0.24–1.18)	0.56 (0.27–1.17)	0.78 (0.25–2.39)
15 years old	0.63 (0.45–0.89)	0.62 (0.29–1.34)	0.68 (0.30–0.51)	0.53 (0.17–1.69)
14 years old	0.44 (0.31–0.61)	0.21 (0.09–0.51)	0.26 (0.14–0.51)	0.21 (0.09–0.49)

Sexual Identity^d^				
Heterosexual	1.00 (Referent)	1.00 (Referent)	1.00 (Referent)	1.00 (Referent)
Gay/Lesbian	1.04 (0.58–1.89)	1.64 (0.62–4.33)	1.81 (0.93–3.51)	1.04 (0.35–3.08)
Bisexual	1.46 (1.03–2.07)	2.27 (1.22–3.85)	2.10 (1.05–4.19)	0.86 (0.34–2.22)
Questioning/Other	0.98 (0.76–1.26)	1.10 (0.59–2.05)	0.63 (0.28–1.13)	0.35 (0.13–0.89)

**NOTE:** Sex, race/ethnicity, age, and sexual identity were used as covariates to estimate adjusted odds ratios.

a Current reflects past 30-day use of each product.

b Binary indicator of self-reported experiencing food insecurity. Yes reflects not reporting “never” to the question: “ During the COVID-19 pandemic, how often did you go hungry because there was not enough food in your home?”

c For race/ethnicity, Hispanic ethnicity does not consider racial category. Non-Hispanic “Other” reflects those who identified as American Indian/Alaskan Native, Asian, Native Hawaiian/Other Pacific Islander, or Multi-Racial, but did not identify as Hispanic/Latino. For this “NH” is “non-Hispanic.”

d Questioning includes responses: “I describe my sexual identity some other way” or “I am not sure about my sexual identity (questioning)”.

### 3.3 Daily tobacco use

As seen in Table 5, experiencing homelessness was associated with greater risk for daily, relative to non-daily, use of e-cigarettes (aOR: 2.66; 95 % CI: 1.17–6.06), cigarettes (aOR: 10.94; 95 % CI: 3.11–38.49), and cigars (aOR: 5.23; 95 % CI: 1.80–15.16), controlling for covariates. No statistical difference in risk for daily (relative to non-daily) smokeless tobacco use was observed between youth who experienced homelessness and those who did not (aOR: 2.48; 95 % CI: 0.51–12.16), controlling for covariates.

**Table 5:** Association between economic disparities and daily tobacco use among high school students in the United States, Spring 2021; Adolescent Behaviors and Experiences Survey (ABES).

	Daily Electronic Cigarette Users N = 6,455	Daily Cigarette Smokers N = 6,775	Daily Cigar Smokers N = 6,841	Daily Smokeless Tobacco Users N = 6,843
	Adjusted Odds Ratio (95 % CI)			
	*Model 1: Experiencing Homelessness and Daily Use*			
Homelessness^a^				
No	1.00 (Referent)	1.00 (Referent)	1.00 (Referent)	1.00 (Referent)
Yes	2.66 (1.17–6.06)	10.94 (3.11–38.49)	5.23 (1.80–15.16)	2.48 (0.51–12.16)

	*Model 2: Food Insecurity and Daily Use*			
Food Insecurity^b^				
No	1.00 (Referent)	1.00 (Referent)	1.00 (Referent)	1.00 (Referent)
Yes	1.57 (1.07–2.31)	1.59 (0.68–3.70)	1.57 (0.48–5.20)	2.82 (0.65–12.12)

**NOTE:** We conducted a multinomial logistic regression to preserve weights and sampling frame. The base (referent) outcome for these analyses was nondaily use (1–29 days/month) and the comparison was daily use (30 out of 30 days/month) use; we did not report comparisons for non-users.

**NOTE:** Sex, race/ethnicity, age, and sexual identity were used as covariates to estimate adjusted odds ratios.

^c^Models controlled for sex, race/ethnicity, and sexual identity. For race/ethnicity, Hispanic ethnicity does not consider racial category. Non-Hispanic “Other” reflects those who identified as American Indian/Alaskan Native, Asian, Native Hawaiian/Other Pacific Islander, or Multi-Racial, but did not identify as Hispanic/Latino. Questioning includes responses: “I describe my sexual identity some other way” or “I am not sure about my sexual identity (questioning).”

a Binary indicator of self-reporting homelessness. Participants were asked: “During the past 30 days, where did you usually sleep?”

b Binary indicator of self-reported experiencing food insecurity. Yes reflects not reporting “never” to the question: “ During the COVID-19 pandemic, how often did you go hungry because there was not enough food in your home?”

Food insecurity was associated with greater risk for daily, relative to non-daily, use of e-cigarettes (aOR: 1.57; 95 % CI: 1.07–2.31), adjusting for covariates. No statistical difference in risk for daily (relative to non-daily) cigarette smoking (aOR: 1.59; 95 % CI: 0.68–3.70), cigar smoking (aOR: 1.57; 95 % CI: 0.58–5.20), or smokeless tobacco (aOR: 2.82; 95 % CI: 0.65–12.12) use was observed, controlling for covariates (See Table 5).

## 4 Discussion

This study found that two measures of economic inequality (experiencing homelessness and food insecurity) were associated with greater odds of current and daily tobacco use among a nationally representative sample of high school students in 2021. To our knowledge, this is the first study to link experiencing homelessness and food insecurity with the daily smoking of cigarettes and cigars in a nationally representative sample of youth; similar findings have been observed using state-level data (Robson et al., 2017). Our findings suggest that the economic disparities in tobacco use, particularly combustible tobacco smoking, observed in national samples of adults in the US (Cornelius et al., 2023, Cornelius et al., 2022) are similarly observed among adolescents (Kim-Mozeleski and Pandey, 2020). Consistent with public health theory, our findings must be interpreted with consideration for both the downstream outcomes (i.e., tobacco use) as well as the upstream determinants (i.e., economic inequalities) of those outcomes.

Comprehensive tobacco control efforts that incorporate prevention, cessation, and regulatory action (King et al., 2014) are needed to address the economic determinants of tobacco use among youth. Our findings showed that a sizable proportion of youth experiencing homelessness reported current use of each tobacco product, from 12.1 % using smokeless products to 37.3 % using e-cigarettes in the past 30-days, compared to between 1.4 % and 15.0 % among youth with stable housing. Preventing the onset and continuation of tobacco use will require an in-depth understanding of the social-cognitive determinants of tobacco initiation and use among youth experiencing economic disparities. Individual-level determinants of tobacco use among youth include harm perceptions, social norms, and self-efficacy (Thomas et al., 2015, Kelder et al., 2021, Mantey et al., 2024); each addressable through evidence-based interventions, such as mass media campaigns and school-based programs.

However, per the CDC’s comprehensive tobacco control framework, direct intervention targeting individual level determinants must be accompanied by addressing environmental factors (King et al., 2014). On average, youth from economically disadvantaged backgrounds encounter more pro-tobacco social norms (e.g., higher adult use) (Cornelius et al., 2023, Cornelius et al., 2022) and messages (e.g., POS marketing) (Glasser and Roberts, 2021, Ma et al., 2022). Consequently, our findings indicate the need for concerted efforts to reduce expose to pro-tobacco environments. Existing efforts to reduce exposure to pro-tobacco messages and norms that disproportionately impact low-income youth include restrictions of tobacco retail outlets and POS marketing within geographic proximity of schools and increasing access to cessation resources for low-income youth who use tobacco (East et al., 2021). Future research will be needed to directly inform the most appropriate interventions needed to address economic disparities in youth tobacco use.

Youth who experienced homelessness were significantly more likely to report daily use of e-cigarettes, combustible cigarettes, and cigars, indicating a need for cessation services tailored to the needs of these youth. However, promoting tobacco cessation among adolescents experiencing homelessness or who are food insecure may prove difficult as current interventions do not adequately consider nor address barriers to adolescent tobacco cessation (Vallata et al., 2021). In fact, most tobacco cessation interventions for adolescents occur within school settings (Bold et al., 2022). However, adolescents experiencing homelessness or who are food insecure are more likely to be absent from school than their counterparts, which could contribute to inequities in adolescent tobacco control efforts (Mills et al., 2022). There is a need to explore innovative tobacco cessation strategies that can reach adolescents in and outside school settings. For instance, in the US, nearly all adolescents aged 13 to 17 have access to a smartphone (95 %) (Vogels et al., 2022), and smartphone-based interventions are efficacious for smoking cessation when combined with pharmacotherapy (Guo et al., 2023). Further, evidence shows that wide-scale dissemination of smartphone apps for smoking cessation reduces tobacco-related health disparities and contributes to healthcare savings (Nghiem et al., 2019). However, few smartphone-based interventions are available for adolescents. Given the increased emphasis on promoting equity in tobacco control efforts (Mills et al., 2022), more attention is needed to understand how health promotion specialists can promote evidence-based cessation assistance to hard-to-reach and vulnerable adolescents.

While addressing tobacco use among low-income youth is essential, so too is the need to reduce the number of youth experiencing homelessness (Mialon, 2020) and food insecurity (Naumann et al., 2024). As observed in this study, a substantial proportion of youth experienced economic disparities, including nearly 1 in 4 high school students (23.7 %) experiencing food insecurity in 2021. Economic indicators are consistently the strongest correlate of tobacco use among adults in national samples (Cornelius et al., 2023, Cornelius et al., 2022). Similarly, the World Health Organization (WHO) has noted that impact of housing access as an essential determinant of health and inequities (Mialon, 2020). Factors related to economic inequality (e.g., stress) have been proposed as a possible for the observed association between economic disparities and tobacco use, including among youth and young adults (Clendennen et al., 2021, Mantey et al., 2022).

Interventions and policies specifically designed to reduce the prevalence of adolescent food insecurity are relatively understudied; however, a recent analysis found that greater investment in addressing food insecurity corresponded with lower substance use rates among adults in the US (Naumann et al., 2024). School meal programs are the only policy or program that has consistently been examined with adolescent food insecurity (Roustit et al., 2010). School meal programs, consisting of the National School Lunch Program and the School Breakfast Program, are operated through the United States Department of Agriculture and provide free and/or reduced-price lunch and breakfast options for eligible school-aged children at public and nonprofit schools in the US. Some states, such as Vermont, have piloted universal free school meal programs (meaning free lunch is available to all students regardless of income status), which have demonstrated positive, multi-level, and multi-domain results on child food insecurity, academic and other outcomes (Taylor et al., 2020) however, there has been minimal exploration of programs specific to adolescents. Thus, more work is needed to understand the determinants of adolescent food insecurity better and develop and evaluate adolescent-specific food insecurity mitigation programs. Continued investigation will be needed to determine if this is similarly observed during adolescence.

This study has several limitations. First, we analyzed self-reported data. Consequently, our estimates are subject to recall and response bias. Second, our data are cross-sectional; thus, the temporal relationship between all variables in our study cannot be investigated. Third, the prevalence of housing instability was relatively rare (∼1.8 %). As such, the estimates for this variable are limited by statistical power. To this point, the relatively rare prevalence of this outcome limited the examination of interactions and effect modification. Additionally, the relationship between housing instability and daily smokeless tobacco use did lack statistical significance, however, this may be an artifact of low statistical power as there was a significant association with current use and the estimated effect for daily use (aOR: 2.48) was similar to that of the other non-combustible product (e-cigarettes; aOR: 2.66). Future research powered to investigate the intersectional influences on economic disparities and tobacco use during adolescence. Fourth, our findings need replication as the data used for this study was collected during the COVID-19 pandemic (Robson et al., 2017). As the pandemic had plausible impacts on tobacco use (Clendennen et al., 2021) and economic conditions, continued surveillance of economic disparities in adolescent tobacco use is needed. And fifth, data were not collected on possible confounding variables that may impact the precision and statistical significance of the associations observed in this study, such as tobacco marketing and advertising, parent and peer tobacco product use, and retail access to tobacco products.

Despite these limitations, we report important findings for understanding adolescent tobacco use during the COVID-19 pandemic. Youth experiencing homelessness and food insecurity remain a significant public health problem with serious downstream issues, including tobacco use and dependence. Our results suggest that economic disparities in tobacco use observed in adults are also present among adolescents. As a result, interventions focused on reducing the impact or scope of economic disparities may have a “downstream” impact of preventing and reducing youth tobacco use in the United States. Future research is needed to develop a more comprehensive understanding of the economic determinants of tobacco use specific to youth.

## Funding Statement

Research reported in this presentation was supported by grant number [1 R01 CA242171-01] from the National Institutes of Health (NIH). The content is solely the responsibility of the authors and does not necessarily represent the official views of the National Cancer Institute or the National Institutes of Health.

## Author Disclosure

No disclosures to report.

## CRediT authorship contribution statement

**Dale S. Mantey:** Writing – original draft, Writing – review & editing, Methodology, Formal analysis, Conceptualization. **Kathryn M. Janda-Thomte:** Writing – original draft, Writing – review & editing. **Adam C. Alexander:** Writing – review & editing, Formal analysis, Conceptualization. **Onyinye Omega-Njemnobi:** Writing – review & editing, Formal analysis. **Steven H. Kelder:** Writing – review & editing, Project administration, Methodology.

## Declaration of competing interest

The authors declare the following financial interests/personal relationships which may be considered as potential competing interests: Dr. Mantey was a consultant for the State of Minnesota in its case against Juul Labs and Altria. No other conflicts are reported.

## Data Availability

Data will be made available on request.
